# Psychometric Hepatic Encephalopathy Tests and Electroencephalogram Results Among Cirrhotic Patients

**DOI:** 10.3390/medicina60111861

**Published:** 2024-11-14

**Authors:** Alaa Aboud Mohamed, Mostafa M. Elkholy, Ola O. Mangoud, Ahmed R. N. Ibrahim, Marwa O. Elgendy, Ali M. Abdel Fattah

**Affiliations:** 1 Department of Tropical Medicine, Faculty of Medicine, Beni-Suef University, Beni-Suef 62521, Egypt; alibeltagi@med.bsu.edu.eg; 2 Department of Clinical Neurophysiology (Neurodiagnostics and Research Centre), Faculty of Medicine, Beni-Suef University, Beni-Suef 62521, Egypt; mostafaelkholy@med.bsu.edu.eg; 3 Department of Psychology, Minia University, Minia 61519, Egypt; ola.mangoud@mu.edu.eg; 4 Department of Clinical Pharmacy, College of Pharmacy, King Khalid University, Abha 61421, Saudi Arabia; aribrahim@kku.edu.sa; 5 Department of Clinical Pharmacy, Beni-Suef University Hospitals, Faculty of Medicine, Beni-Suef University, Beni-Suef 62521, Egypt; 6 Department of Clinical Pharmacy, Faculty of Pharmacy, Nahda University (NUB), Beni-Suef 62764, Egypt

**Keywords:** psychometric testing, EEG, hepatic encephalopathy

## Abstract

*Background and Objectives*: Patients with cirrhosis who seem normal during physical examinations may still have abnormalities in their electroencephalogram (EEG) or show pathological results in neuropsychological tests. This study aimed to investigate the progression of minimal hepatic encephalopathy, its effects on quality of life, its prognostic value, and its significance for daily functioning. *Materials and Methods*: This study involved 50 patients with confirmed cirrhosis (28 Child A, 12 Child B, 10 Child C) who were assessed for psychological symptoms and underwent several tests: the Minimal Mental State Examination (MMSE), the Letter Cancellation Test, the Digit Symbol Coding Test, and EEG. *Results*: showed that 40% of patients exhibited neuropsychiatric symptoms, with somatization being the most common at 96%. The MMSE revealed cognitive impairment in 48% of patients. In the Letter Cancellation Test (LCT) (total error), 80% of patients had organic disorders, and 24% showed affections with (LCT) (completion time). The Digit Symbol Coding Test results showed affection in 28% of patients. Significant EEG changes were observed in patients with Child C cirrhosis. Patients with portal hypertension (including varices and variceal bleeding), liver cell failure symptoms (such as ascites, lower limb edema, and bleeding tendency), as well as those who smoke, or obese, or have hyperlipidemia, all displayed notable EEG and psychological test abnormalities, making them more likely to develop hepatic encephalopathy. *Conclusions*: psychological testing and EEG changes are effective in detecting minimal hepatic encephalopathy, with a higher incidence in Child C patients compared to those in Child A and B.

## 1. Introduction

After ruling out all other known brain disorders, patients with liver failure and portosystemic shunting are diagnosed with hepatic encephalopathy (HE), a neuropsychiatric condition. HE affects 30 to 45% of patients with cirrhosis, and its severity indicates a poor prognosis and high mortality. HE is categorized as either overt or covert based on clinical and neuropsychological tests. Overt hepatic encephalopathy (OHE) refers to HE that is clinically evident, In contrast, covert hepatic encephalopathy (CHE), previously known as minimal or subclinical HE, describes mild HE that is not clinically obvious and is detected only through specialized cognitive testing [1].

The concept of minimal hepatic encephalopathy (mHE) emerged when experts noticed that some cirrhosis patients appeared normal during clinical exams but showed abnormalities on simple neuropsychological tests or electroencephalograms (EEGs). Subsequent research has explored the clinical course of mHE, its impact on quality of life, its prognostic significance, and its effect on daily activities, such as the ability to operate dangerous machinery or drive a vehicle [2].

Various neuropsychological tests have been developed to identify MHE, including computerized tests like inhibitory control and critical flicker frequency tests, psychometric assessments, and electrophysiological evaluations. The Psychometric Hepatic Encephalopathy Score (PHES), a widely validated paper-and-pencil test battery, is recommended as the best clinical tool for detecting MHE. The PHES score is influenced by the Digit Symbol Test (DST), Number Connection Tests (NCTs) A and B, the Serial Dotting Test (SDT), and the Line Tracing Test (LTT). This battery evaluates several cognitive domains linked to the neuropsychological deficits seen in MHE. The tests are applicable across cultures and are easy for healthcare professionals to perform [3].

Electroencephalography (EEG) has long been used to diagnose hepatic encephalopathy by measuring cortical brain activity. Although this test does not require patient cooperation, other CNS conditions, medications, and metabolic disorders can influence the results. Spectral, computer-assisted EEG analysis is recommended because outcomes can vary depending on the evaluator. Some studies have reported abnormal EEG patterns in up to 85% of patients with minimal hepatic encephalopathy who show no clinical signs of encephalopathy. However, there is ongoing debate about the role of EEG as the primary diagnostic method for MHE [4].

This study aimed to assess the clinical course of minimal hepatic encephalopathy, its impact on quality of life, its prognostic value, and its importance in daily functioning.

## 2. Materials and Methods

The study was conducted in the tropical ward between March and July 2023. A total of 50 patients, aged 18 to 50 years, were included in the study. All participants had a confirmed diagnosis of cirrhosis but showed no obvious symptoms of hepatic encephalopathy at the time of testing (Western Haven grade 0). These patients were recruited from the outpatient clinic of the Department of Tropical Medicine, where they were diagnosed with cirrhosis based on clinical history, serological tests, radiological imaging, and, where available, liver histology.

### 2.1. Inclusion Criteria

- Liver cirrhosis
- Age 18 to 50
- At least an intermediate level of education.

### 2.2. Exclusion Criteria

- Hepatocellular carcinoma
- Use of neuropsychiatric medications
- End-stage disease in other organs (CNS, CVS, or renal)

### 2.3. All Patients Underwent the Following Assessments

1. Comprehensive history-taking: including psychological symptoms.
2. Laboratory tests: liver function tests, lipid profile, kidney function tests, and blood sugar levels.
3. Imaging: abdominal ultrasonography.
4. Upper endoscopy.
5. Psychological tests:
  5.1. Mini Mental State Examination (MMSE): Developed by Folstein et al. (1975) and translated by Elsabwah [5]. The test showed a performance reliability coefficient of 0.69 after an 18-day retest period and an external standard validation coefficient of 0.61 when correlated with the comprehension test level of the Wechsler Adult Intelligence Scale.
  5.2. Selective visual attention tests: One of the cancellation tests developed by Elsabwah, including the Letter Cancellation Test (LCT). These tests are designed to assess various functions of the prefrontal cortex, such as information processing speed, attention span, and executive function. The LCT showed a performance reliability coefficient of 0.89 after an 18-day retest and an external standard validation coefficient of 0.79 when correlated with the Digit Cancellation Test (DCT).
  5.3. Number Symbol Coding Test: Compiled by Wechsler (1981) [6] and translated by Malika & Ismail (1996) [7]. This test assesses prefrontal cortex functions, including information processing speed, attention span, and functional operation. The reliability coefficient for this test was 0.81 after an 18-day retest, and its external standard validation coefficient was 0.76 when correlated with the DCT.
  5.4. Symptom Checklist 90-Revised (SCL-90-R): Published by Derogatis et al. (1973) and translated by Al-Buhairi (2005) [8]. The reliability coefficient for performance on the SCL-90-R was 0.91 after an 18-day retest period, and the internal standard validation coefficient was 0.59 when correlated with the Arabic Mental Health Scale, prepared by Abd Elkhalek (2016) [9].
6. EEG:

EEG recordings were obtained using 19 gold disc electrodes placed on the scalp according to the international 10/20 system, with reference and ground electrodes on the forehead. Electrode impedance was kept below 5 kohms. The raw EEG signals were collected using the Natus Neurowork EEG system (Nicolet EEG Amplifier V32) (Natus Medical, Pleasanton, CA, USA) with a frequency range of 1 to 70 Hz and a sampling rate of 512 Hz. During the 20 min recording, the patient laid supine, relaxed, alert, and with eyes closed in a quiet environment. A technician monitored the session to ensure signal quality, minimize artifacts, and maintain patient alertness.

The EEG data were imported into NeuroGuide software (Deluxe 3.2.1, Applied Neuroscience, London, UK) for analysis. Recordings were referenced to the relevant auditory reference, filtered within a 1 to 30 Hz range, and digitized at a sampling rate of 256 Hz. A manual selection process was used to obtain 2 s epochs over a total duration of 3–4 min, with segments containing visible artifacts excluded. Only records with a split reliability of >90% and test/retest reliability of >95% were accepted for subsequent spectral analysis [10].

The selected EEG segments underwent power spectrum analysis using a fast Fourier transform (FFT) with a 25% sliding window, as per the method of Kaiser and Sterman, to reduce the FFT window effect [10].

This analysis provided average power spectrum values for different frequency bands at the 19 recording locations. The frequency bands used included the following [11]:

Delta (1–3.5 Hz)

Theta (4–7.5 Hz)

Alpha (8–13 Hz)

Beta (14–30 Hz)

The overall absolute and relative power for each of these four frequency bands was averaged across 17 electrodes (excluding FP1 and FP2). Global peak alpha frequencies were also averaged from the same electrodes. The spectral power ratio was calculated by dividing the mean absolute power value of the delta and theta bands by the value of the alpha and beta bands.

## 3. Results

Diagnosis of cirrhosis is based on clinical history, serological tests, imaging, and liver histology, if available. This study included 50 patients with cirrhosis (28 Child A, 12 Child B, and 10 Child C). As shown in Table 1, 40% of our patients had neuropsychiatric symptoms, the most common of which were somatization (96%), obsessive-compulsive (88%), and hostility (84%), followed by depression (80%), anxiety (76%), paranoid ideation (68%), psychoticism (68%), interpersonal sensitivity (64%), and phobic anxiety (32%).

Results of neuropsychological tests reveal a strong relationship with the patients under investigation; in the Mini Mental State Examination (MMSE), 24 patients (48%) display cognitive impairment (20 of them demonstrated mild cognitive impairment, 4 displayed moderatecognitive impairment). In the Letter Cancellation Test (LCT) (total error), 40 (80%) patients have organic disorders, and for LCT (completion time), 12 (24%) patients showed affection. Digit Symbol Coding Test results showed affection of 14 (28%) of patients as shown in Table 1 and Table 2.

There were significant-an extremely important changes in Global relative delta power (GRPD), global relative theta power (GRPT), global relative alpha power (GRPA), and global relative beta power (GRPB) in Child C as well as in Child A and B, However, there were no significant changes in global absolute delta power (GAPD), global absolute alpha power (GAPA), global absolute beta power (GAPB), slow power ratio (delta + theta)/fast (alpha + beta), or global highest frequency alpha e (GAPF). Additionally, there were significant EEG changes in global absolute theta wave power (GAPT), which were more significant in Child C, as shown in Table 3.

Table 4 demonstrates that psychological symptoms (somatic, OCD, INT, anxiety, paranoia, and psychosis) were more common in Child C cirrhotic patients than in Child B and A cirrhotic patients and that psychological testing is more meaningful in Child C cirrhotic patients.

Table 5 demonstrates significant EEG changes in patients with the following characteristics: smoking, edema, ascites varices, variceal bleeding, collaterals, bleeding tendency, obesity, and hyperlipidemia. As a result, cirrhotic patients with these characteristics have been considered more risky to develop hepatic encephalopathy.

Table 6 demonstrates significant psychological test abnormalities in patients with the following characteristics: smoking, edema, ascites varices, variceal bleeding, collaterals, bleeding tendency, and obesity hyperlipidemia. As a result, cirrhotic patients who exhibit these traits are predictors of minimal hepatic encephalopathy and are more likely than others to experience the onset of hepatic encephalopathy.

## 4. Discussion

Minimal hepatic encephalopathy (MHE) is the mildest form of hepatic encephalopathy (HE) and is characterized by subtle motor and cognitive impairments that negatively affect health-related quality of life [12]. While cirrhotic patients with MHE typically present with normal neurological and mental status during clinical examinations, they exhibit measurable neuropsychological deficits [13].

MHE refers to subtle changes in cognitive functions, electrophysiological parameters, cerebral neurochemical/neurotransmitter balance, cerebral blood flow, metabolism, and fluid homeostasis in patients with cirrhosis who do not meet the clinical criteria for a diagnosis of HE [14]. These neurocognitive abnormalities mainly impact attention, information processing speed, motor skills, and coordination, but are not detectable through standard neurological examinations. Importantly, these cognitive issues occur independently of sleep disturbances or overall intelligence levels [15].

A previous study reported that psychometric tests are useful in diagnosing HE [16].

The exact prevalence of MHE in patients with portal hypertension is unknown, but it has been reported to affect between 20% and 84% of cirrhotic patients, depending on the diagnostic methods and criteria used [17].

The wide range in prevalence is influenced by factors such as prior episodes of overt hepatic encephalopathy (OHE), severity of liver disease, age, the presence of esophageal varices, and surgical portosystemic shunts. Patients with MHE tend to be older, and more likely to have alcohol-induced cirrhosis, a history of OHE, more advanced liver disease, and esophagogastric varices [18].

EEG is a highly effective tool for diagnosing HE in research settings. HE is associated with a decreased mean frequency of brain electrical activity, with the diagnostic sensitivity of this finding ranging from 43% to 100% in various studies [19].

The results indicated that 40% of our patients experienced neuropsychiatric symptoms, with somatic symptoms, obsessive-compulsiveness, and hostility being the most common, followed by depression, restlessness, paranoia, psychosis, and obsessive anxiety, as reported by the study of Alemam A et al. [20]. Neuropsychiatric symptoms were significantly present in 40% of the patients. In the Mini Mental State Examination (MMSE), 24 patients (48%) showed cognitive impairment according to neuropsychological testing. Of these, 20 had mild cognitive impairment, while 4 displayed moderate impairment. Additionally, the Letter Cancellation Test (LCT) revealed that 40 patients (80%) had organic disorders based on total errors, and 12 patients (24%) showed impairment based on completion time. The Digit Symbol Coding Test results indicated impairment in 14 patients (28%), aligning with the study of Alemam A et al.’s findings that 80% of patients had positive psychometric test results [20].

Alemam A et al., 2016, also noted that minimal HE was more prevalent in patients with Child B and C than in those with class A, as evidenced by EEG changes. Our study supports these findings, revealing significant EEG changes in all patients, with more pronounced changes in those with Child C and B than in class A [20]. However, this study contrasts with the study of El-sherif et al., which found that abnormal EEG traces were not linked to any liver function-related variables [21].

In line with the 2018 study by El-sherif A et al., which showed that low psychometric test scores were associated with functional impairment, our work found that psychological symptoms (including somatic symptoms, obsessive-compulsive behavior, interpersonal sensitivity, depression, anxiety, paranoia, and psychosis) were more prevalent in patients with Child C cirrhosis compared to those with class B or A cirrhosis. Moreover, psychological testing abnormalities were more significant in Child C cirrhotic patients than in those with class B or A [21].

Higher Child–Turcotte–Pugh (CTP) classification was associated with lower scores more frequently: 13.3% in Child A, 40.0% in Child B, and 46.7% in Child C. A study by Alemam A et al. also found a strong correlation between the severity of liver disease and psychological test results [20]. The study also indicated that a child’s score, along with factors like a tendency to bleed, edema, ascites, varices, variceal hemorrhage, collaterals, and obesity are linked to portal hypertension (e.g., varices, ascites, collaterals, and bleeding varices) and liver decompensation symptoms (e.g., bleeding tendency and lower limb edema). Additionally, hyperlipidemia was significantly correlated with both EEG changes and psychological test results, suggesting that obese cirrhotic patients and those with hyperlipidemia are more likely to develop hepatic encephalopathy (HE) [22].

The study by Wunsch et al. studied 50 cirrhotic patients without overt hepatic encephalopathy (OHE). They found that the Psychometric Hepatic Encephalopathy Score (PHES) had a sensitivity of 57% and a specificity of 97% for diagnosing minimal hepatic encephalopathy (MHE) [23]. In cirrhotic patients, PHES was correlated with the severity of liver disease and EEG results. Patients with impaired EEG had lower PHES scores than those with normal EEG, although the agreement between these two methods was limited.

Also, the study by Duarte-Rojo et al. studied volunteers and cirrhotic patients with and without low-grade OHE. They found that 15% of the 84 cirrhotic patients without OHE had MHE [24].

Li et al. applied the five PHES tests to 146 healthy volunteers and 53 patients with liver cirrhosis. They found that 49.1% of cirrhotic patients had MHE, with a higher proportion in decompensated patients (Child B and C) compared to compensated patients (Child A). The NCT-A and DST tests had a sensitivity of 76.9% and a specificity of 96.3% for diagnosing MHE. The study by Li et al. concluded that PHES is diagnostic for MHE [25].

Gad et al. designed a study to screen for MHE in drivers with liver cirrhosis, finding that 66 out of 139 patients showed evidence of MHE. Significant risk factors for MHE included advanced Child–Pugh grade, hepatitis B virus (HBV) etiology, and smoking [26]. Conversely, Maric et al. found that all cirrhotic patients had EEG changes, with 80% showing signs of MHE. The study used three neuropsychological tests, including the DST, which was most effective in identifying MHE. However, the reliance on only one test (DST) in comparison to the full PHES battery may explain differences in results, suggesting EEG might be more accurate than psychometric tests [27].

Saxena et al. studied 75 non-encephalopathic cirrhotic patients using a battery of tests, including NCT, EEG, and auditory P300 event-related potentials (P3ERPs). They found that 47% of patients had MHE, and 59% of these patients progressed to overt encephalopathy within an average of four months. Multivariate analysis showed that abnormal EEG and psychometric test performance were risk factors for developing overt encephalopathy [28].

Several theories explain the pathogenesis of HE, suggesting that neurotoxic substances like ammonia and manganese may enter the brain in liver failure, leading to morphological changes in astrocytes and neuropsychiatric symptoms [29]. It has been conclusively shown that cognitive functions improve with MHE therapy, which may also enhance patients’ quality of life and delay the onset of OHE [13].

## 5. Conclusions

A total of 40% of cirrhotic patients experience psychological symptoms, which may serve as indicators of minimal hepatic encephalopathy (MHE). Abnormalities in psychological tests and EEG changes can also be used to detect MHE, with a higher incidence found in patients with Child C compared to those in classes A and B. Extra care should be taken with individuals displaying cognitive symptoms, as well as high-risk groups like active drivers, those operating heavy machinery, or patients reporting a decline in job performance.

## Figures and Tables

**Table 1:** Psychological symptoms in studied patients.

	Frequency of Abnormal	
Somatization (SOM)	48 (96%)	“score 60 or more”
Obsessive-Compulsive (O-C)	44 (88%)	“score 60 or more”
Interpersonal Sensitivity (INT)	32 (64%)	“score 60 or more”
Depression (DEP)	40 (80%)	“score 60 or more”
Anxiety (ANX)	38 (76%)	“score 60 or more”
Hostility (HOS)	42 (84%)	“score 60 or more”
Phobic Anxiety (PHOB)	16 (32%)	“score 60 or more”
Paranoid Ideation (PAR)	34 (68%)	“score 60 or more”
Psychoticism (PSY)	34 (68%)	“score 60 or more”
Global Severity Index (GSI)	38 (76%)	“score 60 or more”
Positive Symptoms Distress Index (PSDI)	48 (96%)	“score 60 or more”
Positive Symptoms Total (PST)	20 (40%)	“score 60 or more”
MMSE	24 (48%)	20 mild, 4 moderate cognitive impairment
	“Normal: 24–30, mild cognitive impairment: 19–23, moderate: 10–18, severe < 10”	
DSCT	14 (28%)	“score < 7”
LCT “Total errors”	40 (80%)	“score > 1.8”
LCT “completion time”	12 (24%)	“score > 140 s”

**Table 2:** Psychometric tests in studied patients.

Neuropsychological Measures			
	N = 50		
	Mean ± SD	Cut-off Scores	
Mini Mental State Examination (MMSE)			
(MMSE)	22.24 ± 2.41	18–26	Early-stage dementia
Digit Symbol Coding Test (DSCT)			
(DSCT)	7.95 ± 2.58	10 ± 3	
Letter Cancellation Test (LCT)			
Omission Errors (OM.E. LCT)	3.95 ± 5.84		Organic disorder
False Alarm Errors (FA.E. LCT)	0.32 ± 0.82		
Total Errors (TE. LCT)	4.26 ± 6.04	1.14–4.66	
Completion Time (T. LCT)	123.38 ± 46.74	70.46–236.46	
Perseverations (P. LCT)	0.61 ± 0.22		

**Table 3:** Comparison between Child scores (A, B, C) as regards EEG results.

	Child Score (A, B, C)						χ^2^	*p*-Value
	(1) N = (28)		(2) N = (12)		(3) N = (10)			
	M	SD	M	SD	M	SD		
Global absolute power of delta waves (GAPD)	30.85	50.07	24.82	10.96	20.59	7.01	4.906	0.086
Global absolute power of theta waves (GAPT)	34.68	52.26	29.33	15.72	37.07	16.50	9.374	0.009
Global absolute power of alpha waves (GAPA)	44.64	56.40	34.7	15.94	31.13	14.03	1.433	0.488
Global absolute power of beta waves (GAPB)	113.62	216.18	72.52	49.99	86.73	56.09	31.89	0.087
Global relative power of delta waves (GRPD)	0.31	0.42	0.34	0.45	0.10	0.02	0.08	0.000
Global relative power of theta waves (GRPT)	0.35	0.55	0.36	0.35	0.16	0.03	0.02	0.000
Global relative power of alpha waves (GRPA)	0.90	2.07	0.46	0.46	0.21	0.02	0.04	0.000
Global relative power of beta waves (GRPB)	0.35	0.47	0.31	0.31	0.09	0.07	0.02	0.012
Slow(delta+theta)/Fast(alpha+beta) power ratio Slow/Fast_power_ratio	0.57	0.25	0.56	0.21	0.49	0.19	0.421	0.810
Global alpha e peak frequency (GAPF)	9.52	0.24	9.58	0.14	9.52	0.10	0.326	0.850

**Table 4:** Comparison between different Child scores as regards psychological symptoms and tests.

	Child Score (A, B, C)						χ^2^	*p*-Value
	A (N = 28)		B (N = 12)		C (N = 10)			
	M	SD	M	SD	M	SD		
Symptoms Check List 90-Revised (SCL-90-R)								
Somatization (SOM)	74.57	9.371	71.33	10.03	79.39	8.02	35.12	0.01
Obsessive-Compulsive (O-C)	72.64	8.22	67.33	12.22	80.08	9.27	78.77	0.000
Interpersonal Sensitivity (INT)	61.79	11.16	52.67	15.42	67.44	7.09	88.69	0.000
Depression (DEP)	66.43	9.12	56.50	18.48	67.61	10.01	82.74	0.000
Anxiety (ANX)	71.43	9.30	57.33	15.22	75.12	13.00	94.05	0.000
Hostility (HOS)	79.57	10.12	60.83	20.48	75.50	18.03	85.12	0.000
Phobic Anxiety (PHOB)	56.93	11.25	52.83	27.20	51.56	19.89	86.11	0.000
Paranoid Ideation (PAR)	61.57	11.17	56.50	16.11	72.19	18.01	70.83	0.000
Psychoticism (PSY)	67.43	11.03	54.67	16.26	75.13	14.05	55.95	0.000
Global Severity Index (GSI)	70.43	8.37	59.83	13.14	76.09	11.90	68.60	0.000
Positive Symptoms Distress Index (PSDI)	68.57	7.40	72.83	15.09	78.79	16.09	91.07	0.000
Positive Symptoms Total (PST)	55.21	7.65	46.67	12.94	64.31	10.97	82.74	0.000
Mini-Mental State Examination (MMSE)	22.57	2.13	21.17	2.79	24.96	3.01	29.17	0.01
Digit Symbol Coding Test (DSCT)	8.43	2.55	6.83	2.52	8.20	3.86	42.81	0.000
Letter Cancellation Test (LCT)								
Omission Errors (OM.E. LCT)	5.23	6.55	1.40	1.43	0.00	0.000	34.50	0.000
False Alarm Errors (FA.E. LCT)	0.23	0.59	0.00	0.000	3.00	0.79	48.21	0.000
Total Errors (TE. LCT)	5.46	6.84	1.40	1.43	4.09	3.70	34.36	0.001
Completion Time (T. LCT)	117.64	46.65	138.59	48.39	122.57	41.02	81.39	0.000
Perseverations (P. LCT)	0.62	0.22	0.57	0.22	0.56	0.19	92.629	0.000

**Table 5:** Predictors of EEG parameters results.

Model		Unstandardized Coefficients		R	R Square	t	Sig.	F	Sig.
		B	Std. Error						
1 ^a^	(Constant)	3.244	0.585			5.541	0.000	7.745	0.000
	Smoking status	−0.298	0.055	0.797	0.635 ^a^	−5.387	0.000		
	Obesity	0.168	0.063			2.651	0.011		
	Hyperlipidemia	−0.179	0.066			−2.710	0.010		
	Perispleinic or perihepatic collaterals by US	−0.258	0.083			−3.121	0.003		
	BLEEDING TENDENCY	−0.183	0.080			−2.291	0.027		
	Edema	−0.206	0.097			−2.117	0.041		
	Ascites	0.319	0.089			3.601	0.001		
	History of variceal bleeding	−0.560	0.180			−3.112	0.003		
	EGD	−0.293	0.046			−6.347	0.000		
2 ^b^	(Constant)	8.249	0.622			13.258	0.000	3.689	0.002
	Smoking status	0.160	0.081	0.673	0.454 ^b^	1.967	0.056		
	Hyperlipidemia	0.331	0.072			4.565	0.000		
	Perispleinic or perihepatic collaterals by US	0.218	0.091			2.400	0.021		
	Bleeding tendency	−0.148	0.075			−1.959	0.057		
	Ascites	−0.215	0.117			−1.837	0.074		
	Child score	−0.214	0.100			−2.155	0.037		
	History of variceal bleeding	0.565	0.156			3.629	0.001		
	EGD	0.133	0.058			2.308	0.026		

^a^ Dependent variable: SLOW/FAST. Predictors: (constant), EGD, hyperlipidemia, smoking status, edema, history of variceal bleeding, obesity, perispleinic or perihepatic collaterals by US, bleeding tendency, and ascites. ^b^ Dependent variable: GAPF. Predictors: (constant), EGD, hyperlipidemia, smoking status, history of variceal bleeding, perispleinic or perihepatic collaterals by US, bleeding tendency, ascites, and child score.

**Table 6:** Predictors of psychological test results ^®^.

Model		Unstandardized Coefficients		R	R Square	t	Sig.	F	Sig.
		B	Std. Error						
1 ^a^	(Constant)	−4.705	4.735			−0.994	0.326	15.903	0.000
	Hyperlipidemia	2.511	0.503	0.870	0.756 ^a^	4.998	0.000		
	Perispleinic or perihepatic collaterals by US	3.699	0.682			5.422	0.000		
	Bleeding tendency	3.981	0.740			5.381	0.000		
	Edema	−4.121	0.900			−4.577	0.000		
	Ascites	2.025	1.150			1.760	0.086		
	Child score	1.876	0.556			3.376	0.002		
	History of variceal bleeding	3.607	1.637			2.203	0.033		
	EGD	2.054	0.366			5.606	0.000		
2 ^b^	(Constant)	−15.880	5.439			−2.920	0.006	6.092	0.000
	Hyperlipidemia	2.567	0.733	0.678	0.459 ^b^	3.503	0.001		
	Perispleinic or perihepatic collaterals by US	2.756	0.944			2.920	0.006		
	Ascites	1.619	0.756			2.141	0.038		
	History of variceal bleeding	6.601	1.680			3.930	0.000		
	EGD	1.388	0.525			2.646	0.011		
Letter Cancellation Test (LCT)									
3 ^c^	(Constant)	−4.316	1.148			−3.758	0.001	33.824	0.000
	Bleeding tendency	1.481	0.299	0.876	0.767 ^c^	4.955	0.000		
	Edema	−1.063	0.314			−3.384	0.002		
	Ascites	0.945	0.441			2.140	0.038		
	Child score	1.890	0.258			7.325	0.000		
Symptoms Check List 90-Revised (SCL-90-R)									
4 ^d^	(Constant)	84.145	26.493			3.176	0.003	9.090	0.000
	Hyperlipidemia	−7.628	2.812	0.800	0.639 ^d^	−2.713	0.010		
	Perispleinic or perihepatic collaterals by US	−7.666	3.817			−2.008	0.051		
	Bleeding tendency	13.992	4.140			3.380	0.002		
	Edema	−21.387	5.038			−4.245	0.000		
	Ascites	30.574	6.436			4.750	0.000		
	Child score	13.255	3.109			4.264	0.000		
	History of variceal bleeding	−16.727	9.159			−1.826	0.075		
	EGD	−7.377	2.051			−3.597	0.001		

^a^ Predictors: (constant), EGD, hyperlipidemia, edema, history of variceal bleeding, perispleinic or perihepatic collaterals by US, bleeding tendency, ascites, child score. Dependent variable: MMSE. ^b^ Predictors: (constant), EGD, hyperlipidemia, history of variceal bleeding, perispleinic or perihepatic collaterals by US, ascites. Dependent variable: DSCT. ^c^ Predictors: (constant), edema, bleeding tendency, child score, ascites. Dependent variable: FA.E. LCT. ^d^ Predictors: (constant), EGD, hyperlipidemia, edema, history of variceal bleeding, perispleinic or perihepatic collaterals by US, bleeding tendency, ascites, child score. Dependent variable: (GSI).

## Data Availability

The datasets used and/or analyzed during the current study are available from the corresponding author upon reasonable request.
